# Pacing Strategies of 1500 m Freestyle Swimmers in the World Championships According to Their Final Position

**DOI:** 10.3390/ijerph18147559

**Published:** 2021-07-15

**Authors:** Beatriz Lara, Juan Del Coso

**Affiliations:** 1 Exercise Physiology Laboratory, Camilo José Cela University, 28692 Madrid, Spain; blara@ucjc.edu; 2 Centre for Sport Studies, Rey Juan Carlos University, 28942 Fuenlabrada, Spain

**Keywords:** sports performance, endurance competition, pacing behavior, elite athlete

## Abstract

In 1500 m freestyle swimming races, pacing is generally represented by a parabolic or U-shaped curve indicating that swimming velocity is greatest at the start and the last laps of the race while swimmers maintain an even pace through the middle section of the race. However, there is no information to determine if 1500 m race winners select pacing different to other, less successful swimmers within the same competition. Therefore, this investigation aimed to describe the pacing strategies adopted by 1500 m freestyle competitive swimmers in World Championships (long course), from 2003 to 2019 to determine the most effective pacing to obtain victory or a medal. The official overall and split times for 1500 m freestyle races of the *Fédération Internationale de Natation* (FINA) were obtained from the website of this organization. In total, data of 143 swimming performances (71 male and 72 female) were extracted. With the split times, lap times, and position were calculated across the race. To determine differences in the pacing between best- and worst-ranked finalist, swimmers in each race were divided into four groups based on the final position (1st vs. 2nd vs. 3rd vs. 4–8th). All the lap times of the winners of the race were faster than those of participants classified as 4–8th position for men and women races (*p* < 0.05). However, there were no differences in lap velocity among the different positions achieved at the end of the race when it was normalized by average race velocity. Additionally, there were no differences in the lap-to-lap variability among swimmers with different positions at the end of the race. In summary, both men and women elite swimmers selected parabolic pacing consisting of a fast start in the first lap, an even pace close to their average race velocity in the mid-section of the race (from 50 to 1400 m), followed by an end spurt in the final lap(s). This pattern was very similar in all finalists irrespective of the final position in the race. Hence, the obtaining of a medal in the World Championships was associated to possessing a faster average race velocity rather than a specific pacing profile through the race.

## 1. Introduction

In swimming, overall performance is measured by the time employed to complete a set distance in the water. Hence, all swimming training strategies and preparation routines for the competition are aimed to complete the race in the fastest possible time. In current elite swimming, the margins between winning and losing a race are sometimes narrow and swimming performance is not only influenced by the physical or technical capacities of the swimmer, as pacing across the race may play an important role in the overall result of the race. The importance of pacing in swimming depends on the distance. For example, shorter races (e.g., 50 m) are all-out disciplines covered in about 20 to 35 s, depending on the style, and pacing has little impact on the race time of elite swimmers [1]. However, longer races such as 1500 m freestyle are covered in 14 to 16 min at the highest level and swimmers have to manage their pace to utilize all available resources of energy effectively, to limit premature fatigue [2]. Additionally, the impact of pacing on swimming performance may be more important than in other sports due to low mechanical efficiency and highly resistive properties of water in comparison to land-based sports [3]. To this regard, unlike many cycling or running events, swimmers compete in their own lanes during the whole race and they do not have to compete for positions in particular moments [4]. This means that swimmers are separated from their opponents and the effect of drafting or the change of pace within the competition to gain a position have less impact on swimming performance than in other sports. On the other hand, the effect of the turbulence created by competitors may increase the difficulty while swimming, entailing a reason for a change in pace during the race.

Pacing profiles in swimming are typically characterized by plotting split times or velocity over each lap of the event. In 800 and 1500 m freestyle swimming races, pacing is generally represented by a parabolic or U-shaped curve, indicating that swimming velocity is greatest at the start and the last laps of the race [5,6]. In these distances, swimmers maintain swimming velocity at an even pace close to the average race velocity through the middle of the race, and the second half of the race is completed at a similar pace compared to the first half. By using an analysis of 330 swims of 24 elite male swimmers for the 1500 m freestyle race, it has been suggested that making the first and second laps slower and the penultimate and last laps faster could reduce race time by ~1.4% [6]. In shorter events such as 200 m and 400 m freestyle races, the pattern of lap times was similar between the best and worst swims for finalists [7,8]. However, the final laps are faster in the medalists which suggests that conserving energy at the start, swimming at a consistent pace in the middle sections, and then increase velocity in the latter stages is the most effective pacing strategy [2].

However, there is no information to determine if the winners of 1500 m freestyle races select pacing different to other less successful swimmers within the same competition. Therefore, the aim of this investigation was to describe the pacing strategies adopted by 1500 m freestyle competitive swimmers in World Championships (long course), from 2003 to 2019, to determine the most effective pacing to obtain victory or a medal.

## 2. Materials and Methods

The official overall and split times for 1500 m freestyle races from the World Championships of the *Fédération Internationale de Natation* (FINA) were obtained from the website of this organization (www.fina.org, accessed on 1 February 2021). Only data from the finals were extracted as the aim of this investigation was associated with the study of pacing profiles in winners and medalists in these competitions. Data were obtained from nine different World Championships celebrated between 2003 and 2019, and times were extracted for both men and women competitions. The events were: Barcelona 2003, Montreal 2005, Melbourne 2007, Rome 2009, Shanghai 2011, Barcelona 2013, Kazan 2015, Budapest 2017, and Gwangju 2019. In all these events, the race was performed in FINA validated 50 m lap pools with automatic timing equipment, and the competitions were supervised by FINA officials. Prior to 2003, data of 1500 m freestyle races did not contain split times and, for this reason, their data have not been included in this investigation. Swimmers who did not start or finish or who were disqualified were not included in the analysis. In total, data from 143 swimming performances (71 male and 72 female) were extracted. In men, data were obtained from 9 World Champions, 18 medalists, and 44 swimmers who finished the races between the 4th and 8th positions. In women, the data corresponded to 9 World Champions, 18 medalists, and 45 swimmers who finished the races between the 4th and 8th positions. Using the times, the position of each swimmer at each lap and at the end of the race was calculated. The only data used for this investigation are swimming times and they are publicly available on the internet; the local ethics committee determined that approval was not necessary. Additionally, it was not considered necessary to obtain informed consent from swimmers for use of this publicly accessible information. All procedures were in accordance with the declaration of Helsinki.

The study was designed as observational and descriptive research to describe the pacing tactics selected by finalists in the 1500 m races of the FINA World Championships. To determine differences in the pacing between best- and worst-ranked finalist, swimmers in each race were divided into four groups based on the final position (1st vs. 2nd vs. 3rd vs. 4–8th). The data of swimmers between the 4th and 8th positions were merged as they represent a similar outcome (finalist with no medal) and their data were compared to winners and medalists.

### Statistical Analysis

Initially, the data were electronically extracted from the FINA website and entered into a database designed for the purpose of this research. The data were extracted by one author (B.L.) using an Excel spreadsheet (Microsoft Office 2016, Redmond, WA, USA) and were then checked for accuracy by another author (J.D.C). The mean ± standard deviation for each group and positioning through the race were calculated. Times (in s) and velocity (in m/s) for each lap were calculated from split times and they are presented in absolute values and as a percentage of the average race velocity. For this purpose, average race velocity was calculated as 1500 m/total race time. Afterwards, the time employed during the first and second halves of the race and the fastest and slowest laps were individually calculated. Lap-to-lap variability was calculated as the coefficient of variation in the lap times of the race, excluding the first and last 100 m of the race. This was to analyze the middle section of the race where participants habitually maintain a stable pace. For all these variables, a one-way analysis of variance (ANOVA) was employed to determine differences among groups (1st vs. 2nd vs. 3rd vs. 4–8th). In the case of a significant F-test, a Bonferroni post-hoc analysis was performed to determine differences in pairwise comparisons. The data were analyzed with the statistical package SPSS v 24.0 (SPSS Inc., Chicago, IL, USA). The significance level was set at *p* < 0.05.

## 3. Results

**Figure 1:**
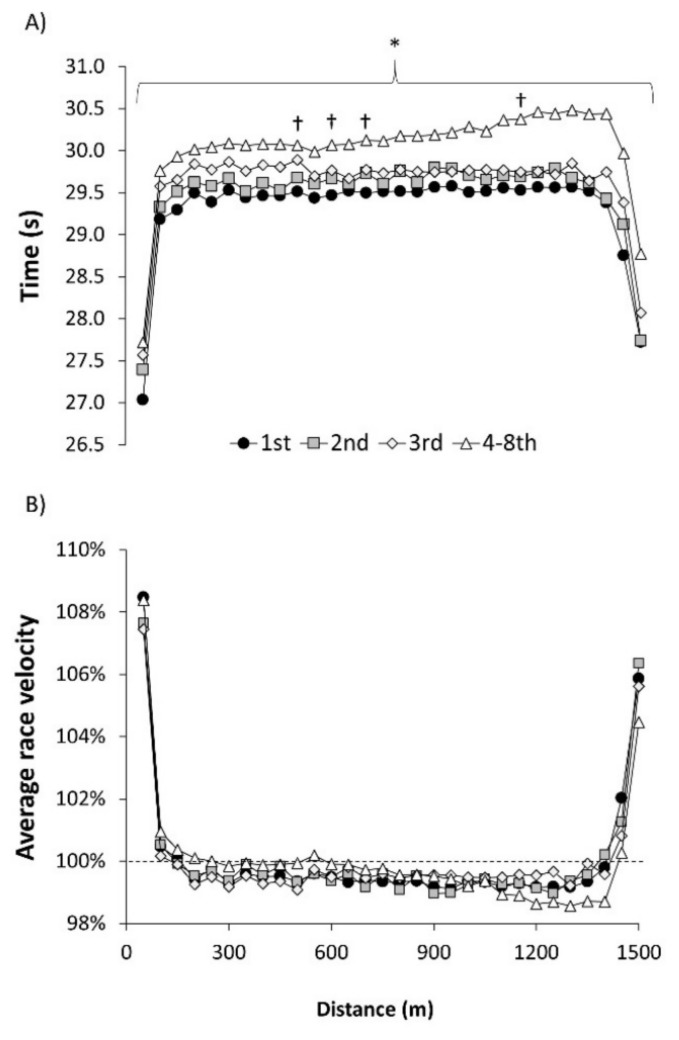
(**A**) Lap times for men swimmers for the 1500 m freestyle races of the World Championships between 2003 and 2019 according to their final position in the race; (**B**) Race velocity in each lap with respect to the average race velocity. (*) Statistically significant difference between 1st and 4–8th positions at *p* < 0.05. (†) Statistically significant difference between 1st and 3rd positions at *p* < 0.05. Error bars have been removed for clarity.

From the 71 swimming performances analyzed in men, the fastest time to complete the 1500 m freestyle race was 14:35:85 obtained in Gwangju 2019, and the worst time was 14:45.94 obtained in Melbourne 2007. The variation between the best time and the worst time to win the World Championships was 1.3%. The upper panel of Figure 1 contains the time employed in each 50 m lap during the 1500 m race according to the position obtained at the end of the race. The association of lap times and distance followed a parabolic curve for all swimmers irrespective of their final position. However, all the lap times of the winners of the race were faster than those of participants classified as 4–8th position in the race (*p* < 0.05). Additionally, the 50 m lap times between 450 and 500 m, 550 and 600 m, 650 and 700, and 1050 and 1100 m were slower in third-place swimmers than the times of the winners (*p* < 0.05). In the lower panel of Figure 1, the swimming velocity in each lap normalized by mean velocity is shown. In the first lap, all participants obtained a velocity between 107.4% and 108.5% of their average race velocity and the pace was reduced in the subsequent laps to a stable pace close to 100.0% of average velocity. Swimming velocity was increased above the average in the last two laps of the race to obtain a final lap between 104.5% and 106.4% of the mean velocity. There were no differences in swimming velocity normalized by average race velocity among the different positions achieved at the end of the race.

Table 1 contains times to complete the two halves of the race, the fastest and slowest lap times, and the percentage differences for these times. In men, the times employed to complete the two halves of the race was higher in 4–8th-position swimmers than those employed by the winners (*p* < 0.05) and the change in time from the first to the second half of the race was also higher (*p* < 0.05). The times of the fastest and slowest laps were also higher in 4–8th-position swimmers than those of winners (*p* < 0.05). However, there were no differences in the lap-to-lap variability among swimmers with different positions at the end of the race.

**Figure 2:**
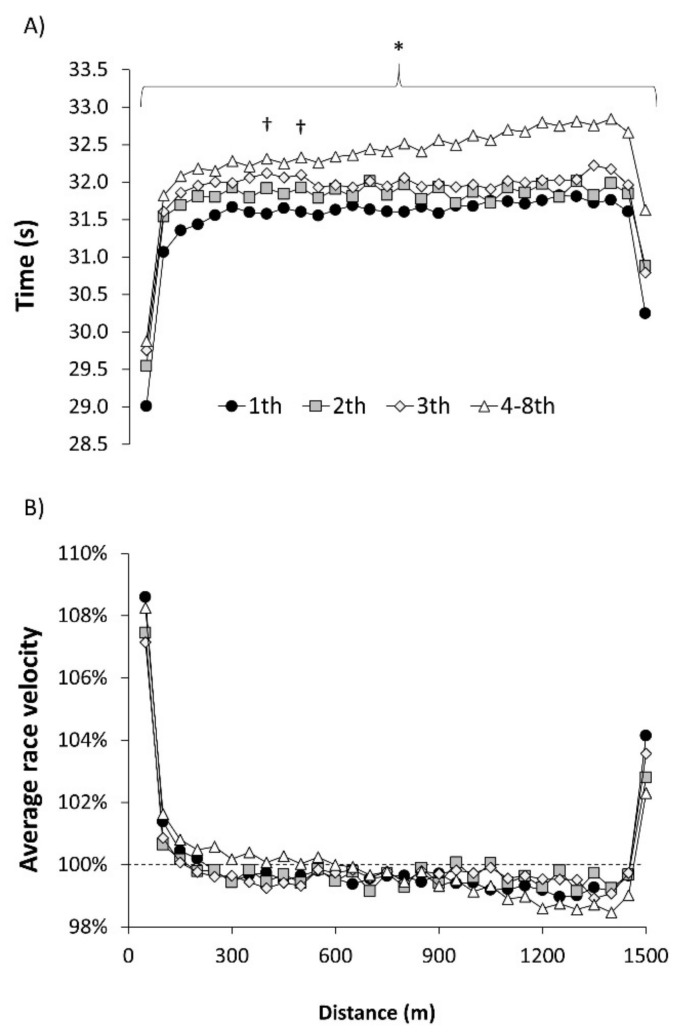
(**A**) Lap times for women swimmers for the 1500 m freestyle races of the World Championships between 2003 and 2019 according to their final position in the race; (**B**) Race velocity in each lap with respect to the average race velocity. (*) Statistically significant difference between 1st and 4–8th positions at *p* < 0.05. (†) Statistically significant difference between 1st and 3rd positions at *p* < 0.05. Error bars have been removed for clarity.

In women, there were 72 swimming performances analyzed and the fastest time to complete the 1500 m freestyle race was 15:25.48 in Budapest 2017 and the worst time was 16:00.41 obtained in Montreal 2005. The variation between the best time and the worst time to win the World Championships was 3.8%. The distribution of lap times across the race distance followed a parabolic curve for all swimmers irrespective of their final position and it was very comparable to the pacing adopted by male swimmers (Figure 2). All the lap times of the winners were faster than those of participants classified as 4–8th position in the race (*p* < 0.05). Additionally, the 50 m lap times between 350 and 400 m and 450 and 500 m were slower in swimmers that ended third than the times of winners (*p* < 0.05). In terms of velocity, female swimmers completed the first lap at 107.1%–108.6% of their average race velocity with a subsequent reduction in swimming velocity until the last lap, which was completed at 102.3–104.1% of the mean velocity. As in male swimmers, there were no between-group differences in mean velocity normalized by average velocity.

**Figure 3:**
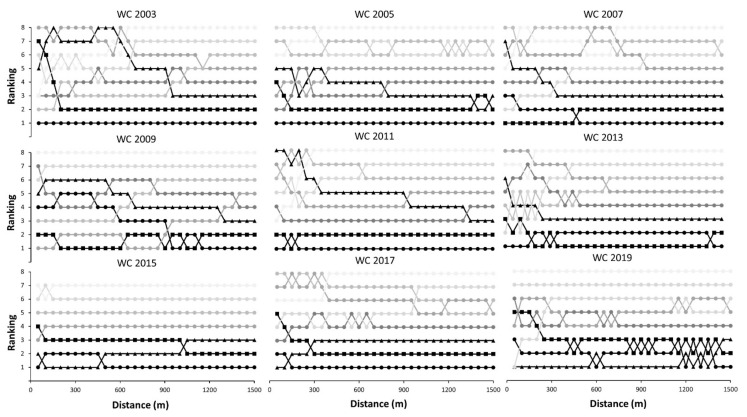
Positioning of male swimmers across 1500 m freestyle races of the World Championships (from 2003 and 2019) according to their final position in the race. WC: World Championship.

**Figure 4:**
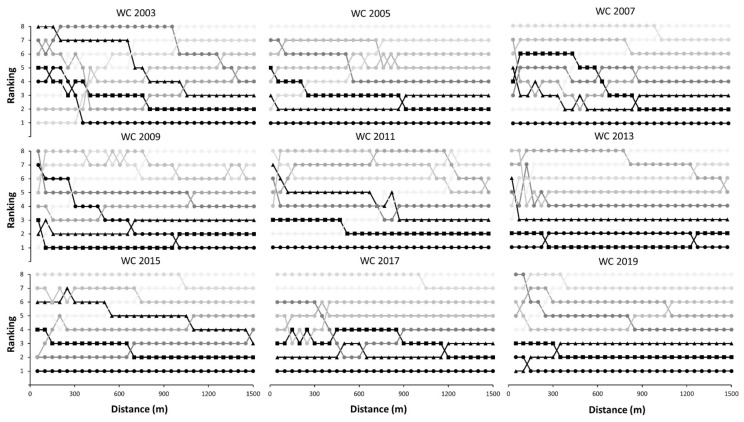
Positioning of female swimmers across 1500 m freestyle races of the World Championships (from 2003 and 2019) according to their final position in the race. WC: World Championship.

Figure 3 and Figure 4 represent the positions of all swimmers during the finals in men and women 1500 m freestyle races, respectively. The winners of the men races were not in a place worse than the 5th position during the race, although the most habitual pattern was that the winner remained within the positions associated with medals in all laps of the race. In women, the winner was always in the first five positions across the race, except in Rome 2009 that occupied the 7th position after the first lap. In 6 out of 9 races in men, the winner was in the first position after 750 m of swimming, while this happened in 7 out of 9 races in women. Overall, no swimmer won the World Championship if they were not in the three-first positions after 600 m of swimming. Regarding obtaining a medal, the tactic was different among medalists but in most cases, they were within the first three positions after 1000 m of racing.

## 4. Discussion

In swimming competitions, the goal is to cover a given distance in the shortest possible time. For this task, the selection of a suitable pacing pattern to use energy in the most efficient manner through the race is a key determinant of success, especially in endurance swimming competitions. The current investigation describes the pacing strategies adopted by 1500 m freestyle competitive swimmers in the World Championships (long course), from 2003 to 2019 according to the final position they obtained in the race. The main outcomes of this investigation are: (a) all swimmers used a pacing described by a parabolic or U-shaped curve with higher speed in the starting lap and at the final laps of the race, irrespective of their final positioning in the race; (b) when swimming velocity is normalized by average race velocity, it is evident that all swimmers chose similar pacing associated with maintained speed close to their mean velocity with all-out final laps; (c) winners are the swimmers with the faster lap times since the first lap (statistically significant differences only from 4–8th-position swimmers) and they swam with a stable pace in the middle section of the race with a lap-to-lap variability between 0.7 (men) and 0.8% (women); (d) to obtain the victory, swimmers had to be in the first 5 positions during the whole race while leading the race after the first half concedes a high likelihood of success in the race; (e) all these characteristics were very similar in men and women 1500 m races suggesting that there is no sex-specific difference in the most successful pacing to obtain victory or a medal in the World Championships. Collectively, all this information suggests that swimmers adopted a similar pacing for the 1500 m races of the World Championships, irrespective of their final position in the race. This pace consisted of a fast start, the adoption of a stable pace at ~1% below their average race velocity, and an all-out final in the last lap(s). However, winners and medalists swam faster since the beginning because their average race velocity was higher than those with a worse final position. These results provide coaches and sports scientists with practical information regarding the most successful pacing to be implemented competition although it shows that the obtaining of a high swimming velocity is more important than pacing for this swimming endurance race.

Previous investigations had established that endurance swimmers competing in long-course pools (i.e., 800 and 1500 m freestyle swimmers) adopted a pace with the greatest velocity at the start and at the last laps of the race accompanied by a stable velocity in the laps between 50 and 1400 m of the race [5,6]. This pacing is different to sprint swimming races where an all-out or near all-out pacing strategy is habitually used [1,9] but similar to open-water endurance competitions [10]. However, this is the first investigation that provides an analysis of pacing profile and positioning across the whole 1500 m race for each of the swimmers, according to their final position in the competition. The study of the pacing of more and less successful swimmers is helpful to understand what the best tactic would be to win a World Championship or to obtain a medal. In this context, it seems that pacing has an important role for 1500 m races as all swimmers chose the same strategy, irrespective of their final performance or their sex (Figure 1 and Figure 2). However, the lack of differences in pacing among more and less successful swimmers suggests that the role of pacing is secondary to the obtaining of a high average swimming velocity throughout the race. In men swimmers, the first lap is covered at ~108.1% of the average race velocity in all swimmers but this represents 1.85 m/s for the winner and 1.80 m/s for the swimmers that obtained the 4–8th position in the race. Similarly, all male swimmers swam from 100 to 1400 m at ~99.6% of their average velocity but it represented 1.70 m/s for the winners and 1.66 m/s the swimmers that obtained the 4–8th position in the race. In women, the starting lap and middle section were covered and at 107.9% and at ~99.6%, respectively, but differences in absolute velocities of winners (1.72 and 1.58 m/s) and the 4–8th-position swimmers (1.67 and 1.54 m/s) are evident. Of note, the worse-ranked swimmers at the end of the race presented a higher change from the first to the second half of the race (Table 1) suggesting a higher level of fatigue in the last laps of the race. Additionally, the only difference pacing difference between male and female swimmers is that men increased their velocity above the mean velocity in the last two laps of the race (Figure 1) while women only did so in the last lap of the race (Figure 2). In summary, the current analysis indicates that the pacing associated to an efficient distribution of energy for the 1500 m freestyle race includes a fast first lap (as the result of the diving start), a stable pace close to the average race velocity for most of the race (from 50 to 1400 m) and a great spurt at the end of race.

Figure 3 and Figure 4 represent the tactics employed by the swimmers during the competition in terms of positioning. Overall, the most employed tactic was the use of a high pace since the beginning of the race to occupy one of the first three positions even after the first 50 m of the race. In fact, in 2 out of 9 men races and 5 out of 9 women races, the winner maintained the first position during the whole race. The manner to obtain a medal varied among competitions, and some swimmers obtained a medal even when they were in the last position in the first laps of the race. However, this is anecdotical as in most cases were within the first three positions after 1000 m of racing. Again, these data suggest that most World Champions and medalist employed a similar tactic consisting of swimming at a fast but stable pace to be within the first positions throughout the race.

The current investigation possesses some limitations that should be discussed to clarify the reach of the study outcomes. First, the analysis presented here is associated with lap times during the 1500 m freestyle races of the World Championships and, therefore, it is not valid to differentiate between swimming and non-swimming components (dive start, turns, and the finish) of the race. However, due to the duration of these races (i.e., >14 min) and the stable pace through most sections of the races, it seems reasonable suggesting that the influence of these non-swimming components was minor for the result of the investigation. Second, swimming velocity is the product of stroke rate and stroke length [11]. However, the current analysis has not investigated whether there is an optimal combination of stroke rate and stroke length to maintain the pace during the race or whether the differences in lap times between best- and worse-ranked swimmers were associated to changes in stroke rate or stroke length. Further investigations with this aim are warranted.

Collectively, this investigation suggests that all swimmers in this swimming endurance event selected an equal pacing consisting of maintained speed through the whole distance plus a fast final lap(s). Hence, the obtaining of a medal in the World Championships was associated to being faster than the rivals since the beginning of the race but with the need of maintaining this pace till the end of the race. From a practical perspective, the development of the capacity of swimming with stable pacing should be obtained through repeated practice and simulated and real competitions [12] as this would maximize the use of energy in a the most effective manner while limiting premature fatigue [2]. To this regard, the capacity to reproduce the pacing irrespective of the conditions of the competition seems to be one of the characteristics of elite swimmers [13]. For this reason, it is recommended that the training plan includes sessions aimed to replicate the pace that will be maintained during a 1500 m swimming competition to aid in the obtaining of a stable pace. Additionally, participating in several races across the season, and particularly in the weeks prior to the most important race of the season, with a posterior analysis and evaluation of the pace maintained, is also recommended to determine which is the faster pacing that a swimmer can maintain during a 1500 m freestyle race. Lastly, the effect of elevated ambient temperature, and altered oxygen content of the air due to altitude and reduced energy availability due to incomplete recovery from a previous competition should be considered when selecting the pacing selected for a 1500 m freestyle race [14].

## 5. Conclusions

1. In the 1500 m freestyle races of the World Championships, all finalists chose similar parabolic pacing during the event characterized by a fast start in the first lap, an even pace close to their average race velocity in the mid-section of the race (from 50 to 1400 m) followed by an end spurt in the final lap(s). This pattern was very similar in both male and female swimmers, and it was irrespective of the final position in the race. However, winners and medalists were faster even in the first lap of the competition and maintained a faster and more stable pacing across the whole race than the remaining participants in the finals;
2. The progressively slower pacing in the worse-ranked finalists, respect to winners, indicates that they were unable to maintain their initial pace during the race due to fatigue. The winners and medalists presented a better management of the pace during the race with no clear signs of a fatigue-induced reduction in the pace;
3. The analysis of positions through the 1500 m race indicates that most World Champions were within the first three positions for the whole race while no swimmer has won the Championship if they were not in the three-first positions after 600 m of swimming.

## Figures and Tables

**Table 1:** Time for the first and second half of the race and the fastest and slowest laps in the 1500 m freestyle races of the World Championships between 2003 and 2019 according to their final position in the race; (*) Statistically significant difference between 1st and 4–8th positions at *p* < 0.05. (†) Statistically significant difference between 1st and 3rd positions at *p* < 0.05.

	Final Ranking	1st Half (s)	2nd Half	1st–2nd Half Change (%)	Fastest Lap (s)	Slowest Lap (s)	Fastest-Slowest Lap (%)	Lap-to-Lap Variability (%)
Men	1st	439 ± 2	440 ± 3	0.3 ± 0.9	26.9 ± 0.5	29.9 ± 0.2	11.2 ± 2.0	0.7 ± 0.1
	2nd	442 ± 4	443 ± 4	0.3 ± 0.5	27.2 ± 0.3	30.1 ± 0.3	10.7 ± 2.7	0.8 ± 0.1
	3rd	444 ± 5	444 ± 4	0.0 ± 1.6	27.3 ± 0.5	30.3 ± 0.3 †	11.2 ± 2.1	1.0 ± 0.3
	4–8th	448 ± 4 *	453 ± 4 *	1.1 ± 1.1 *	27.7 ± 0.4 *	30.8 ± 0.3 *	11.3 ± 1.9	1.0 ± 0.4
Women	1st	471 ± 6	474 ± 7	0.7 ± 0.8	29.0 ± 0.7	32.1 ± 0.6	10.7 ± 2.2	0.8 ± 0.2
	2nd	475 ± 6	477 ± 5	0.4 ± 0.9	29.5 ± 0.6	32.3 ± 0.3	9.2 ± 1.5	0.7 ± 0.2
	3rd	477 ± 4	479 ± 5	0.4 ± 0.7	29.7 ± 0.5	32.5 ± 0.3	9.2 ± 1.7	0.8 ± 0.2
	4–8th	481 ± 5 *	489 ± 6 *	1.6 ± 1.1 *	29.9 ± 0.5 *	33.1 ± 0.4 *	10.8 ± 2.1	1.0 ± 0.4

## Data Availability

The data are publicly available at www.fina.org, accessed on 1 February 2021.
